# The epidemiology of eczema in children and adults in England: A population‐based study using primary care data

**DOI:** 10.1111/cea.13784

**Published:** 2020-11-26

**Authors:** Simon de Lusignan, Helen Alexander, Conor Broderick, John Dennis, Andrew McGovern, Claire Feeney, Carsten Flohr

**Affiliations:** ^1^ Nuffield Department of Primary Care Health Sciences University of Oxford Oxford UK; ^2^ Royal College of General Practitioners Research and Surveillance Centre London UK; ^3^ Unit for Population‐Based Dermatology Research, St John's Institute of Dermatology, Guy's & Thomas' NHS Foundation Trust and King's College London London UK; ^4^ Momentum Data Pendragon House St. Albans UK; ^5^ Pfizer Ltd Tadworth UK

**Keywords:** atopic dermatitis, dermatology, eczema, epidemiology, incidence, primary care

## Abstract

**Background:**

Whilst eczema is a common inflammatory skin condition, we lack contemporary estimates of disease incidence and prevalence across the lifespan.

**Objective:**

To estimate the incidence and prevalence of eczema in children and adults in England and variation by sociodemographic factors (sex, socio‐economic status, ethnicity, and geography).

**Methods:**

We used the Royal College of General Practitioners Research and Surveillance Centre primary care research database of 3.85 million children and adults registered with participating general practitioner practices between 2009 and 2018 inclusive. Eczema incidence was defined as the first‐ever diagnosis of eczema recorded in the primary care record, and eczema prevalence was defined as fulfilment of criteria for active eczema (two eczema records appearing in the primary care record within any one‐year period).

**Results:**

Eczema incidence was highest in infants younger than 1 year (15.0 per 100 person‐years), lowest in adults aged 40–49 (0.35 p/100 person‐years), and increased from middle age to a second smaller peak in people 80 years or older (0.79 p/100 person‐years). Eczema prevalence was highest in children aged 2 (16.5%) and lowest in adults aged 30–39 (2.8%). Eczema incidence was higher in male infants (<2) and male adults older than 70; for all other ages, incidence was higher in females. Eczema was more common in Asian and black ethnic groups than in people of white ethnicity. Higher socio‐economic status was associated with a greater incidence of eczema in infants younger than 2, but the reverse was seen for all other age groups. Both incidence and prevalence of eczema were greater in urban settings and in North‐West England.

**Conclusions and Clinical Relevance:**

Eczema has a bimodal distribution across the lifespan. We observed differences in incidence and prevalence of eczema by ethnicity, geography, sex, and socio‐economic status, which varied in magnitude throughout life.

## 1 INTRODUCTION

Eczema, also known as atopic dermatitis, is a chronic inflammatory skin condition that affects around 200 million people world‐wide.^1^, ^2^ The incidence of eczema has risen significantly over past decades, in particular in high‐income countries.^3^ It most commonly develops in the first year of life, although onset can occur at any age.^4^, ^5^ Eczema usually follows a chronic relapsing–remitting course, and maintaining disease control may require the use of ongoing treatment.^6^

Whilst many eczema‐affected children will have resolution or improvement by late childhood,^4^ a substantial proportion of people will have ongoing eczema into adulthood, and flare ups can occur even after long periods of remission.^5^, ^7^ Itch, discomfort, and visible skin lesions result in disturbed sleep and social embarrassment and affect the quality of life of those affected and their families.^8^ When moderate to severe, the psychological impact in children and adults is often profound.^9^, ^10^, ^11^

In the United Kingdom (UK), prevalence estimates vary widely, especially in adults, and contemporary data on the factors influencing eczema development, such as urban environments, are lacking. Recent questionnaire‐based studies suggest prevalence rates of 2.5%‐15% in adults.^12^, ^13^ Given that the majority of eczema patients are seen and treated in primary care in the UK, databases of electronic health records from general practitioner (GP) practices provide a rich data source from which epidemiological analyses can be derived.^14^ In a recent study using the UK Clinical Practice Research Datalink (CPRD), approximately 500,000 people were identified as having eczema between 1998 and 2015, which scales to a UK prevalence of 10%.^15^ However, this study was designed to assess cardiovascular outcomes in eczema not prevalence per se and only assessed the adult population. Another CPRD study was conducted to examine the incidence of eczema in children between 1997 and 2015 and found the highest incidence in those younger than 2 years of age (15.9 [95% CI 15.7–16.1] per 100 person‐years in males and 11.7 [11.5–11.8] in females) and the lowest incidence in those 5 years of age and older (0.4 [0.3–0.4] per 100 person‐years in males and 0.5 [0.5–0.5] in females).^16^

In this retrospective population‐based study, we set out to provide a contemporary description of the incidence of new onset eczema, and the prevalence of active eczema, in children and adults in England and how these estimates vary by sex, socio‐economic status, ethnicity, and geography.

## 2 METHODS

### 2.1 Study design

We used the Oxford‐Royal College of General Practitioners (RCGP) Research and Surveillance Centre (RSC) database to provide a population‐based sample to calculate eczema incidence and prevalence estimates. The RCGP RSC cohort is drawn from a large network of GP practices distributed across England, providing a representative sample of the English population.^17^ Over the entire study period, the RCGP RSC database contained data from 3.85 million people registered with 293 general practitioner (GP) practices across England.

The RCGP RSC database contains demographic data (including age, sex, ethnicity, socio‐economic status [SES], and rurality), clinical diagnoses, anthropometric measurements (eg body mass index [BMI], laboratory test results and prescriptions, recorded using the Read coding system (a widely used, standardized thesaurus of clinical terms).^18^ UK general practice lends itself to this type of study because it is a registration‐based system (each patient can only be registered with a single GP), it has been computerized since the 1990s, and pay‐for‐performance targets introduced in 2004 have resulted in consistent, high‐quality clinical data entry relating to chronic disease.^19^ Studies using RCGP RSC data have been published across a wide range of diseases, including SARS‐CoV‐2, liver disease, atrial fibrillation, asthma, and diabetes.^20^, ^21^, ^22^, ^23^, ^24^

### 2.2 Study population

All adults and children registered with practices contributing data to the RCGP RCS database between 1 January 2009 and 1 January 2019 were eligible for inclusion in the study. Individuals required at least one year of follow‐up in RCGP RSC, unless under 1‐year‐old. People who opted out of record sharing were excluded (approximately 1.8% of the adult population). The full protocol for the study was pre‐specified and has been previously published.^25^

### 2.3 Definition of eczema

Individuals with eczema were identified using a validated algorithm developed for use with UK electronic health records^26^ and applied in recent UK studies in eczema.^15^, ^16^ The positive predictive value of this algorithm is 90% (95% Confidence interval (CI) 80%–91%) in children and 82% (95% CI 73%–89%) in adults.^26^ In brief, eczema is identified by the presence of one diagnostic code and at least two eczema‐related treatment codes on separate days.

Active eczema was defined as the later of two eczema records appearing within any one‐year period by Silverwood et al^15^ in their study of cardiovascular outcomes in atopic eczema (AE) in primary care. Active AE was then assumed to last for 1 year, unless another AE record appeared, in which case its duration was prolonged for an additional 1‐year period.^15^ We utilized this approach but to signify the onset of active eczema we used the first of two codes (rather than the second) within 1 year as this has shown good agreement with physician‐confirmed onset.^26^

### 2.4 Definition of sociodemographic factors

Eczema incidence and prevalence were stratified by age in children (0–17 inclusive, by year) and adults (age categorized as 18–29, 30–39, 40–49, 50–69, 60–69, 70–79, 80+). To examine variation across other sociodemographic factors when stratified by age, we also defined broader age group categories (<2, 2–11, 12–17, 18–49, ≥50). Ethnicity was extracted from the primary care record and grouped into major ethnic groups: white, black, Asian, mixed, and others.^27^ Socio‐economic status (SES) was defined using the official national deprivation measure: index of multiple deprivation (IMD).^28^ This was calculated at the point of data extraction, using patient postcode, with the resultant scores stratified by deprivation quintile according to the national distribution. Rural/urban classification was defined by patient postcode, using the 2011 Office for National Statistics rural–urban classification.^29^

### 2.5 Statistical analyses

#### 2.5.1 Incidence of eczema

Incident cases were defined as individuals with a first‐ever diagnosis of eczema during the study period. Patients with a diagnosis of eczema prior to the study period were excluded. To increase certainty that an eczema diagnosis was incident, individuals with a diagnosis within one year of registering with a practice were excluded from the incident analysis, unless younger than 1‐year‐old. We calculated age group stratified incidence rates (per 100 person‐years) over the study period, with further stratification within each age category by sex, ethnicity, quintile of IMD, urban/rural classification, and geographical region, by dividing the number of incident patients by the sum of person‐years of follow‐up for the total eligible population over the period of interest. Multivariable‐adjusted incidence rate ratios (aIRR) controlling for age category, sex, ethnicity, quintile of IMD, urban/rural classification, and geographical region were calculated using Poisson regression.

#### 2.5.2 Prevalence of active eczema

We estimated the prevalence of active eczema, overall and by age group, for each calendar year. Prevalent individuals were those who met the definition of active eczema on the 31st December of the year in question. Prevalence was calculated by dividing the number of prevalent individuals by the total number of eligible individuals in the study population on the 31st December of each calendar year. Using data from the most recent year (2018), we estimated the age group stratified prevalence of eczema by sociodemographic factors (sex, ethnicity, IMD, urban/rural classification, and geographical region; and the unadjusted and multivariable adjusted odds of prevalent eczema for the same factors using logistic regression).

All statistical analyses were performed using R statistical package software version 3.4.1 (R Core Team, Vienna, Austria, 2017).

### 2.6 Ethics approval

Study approval was granted by the Research Committee of the RCGP RSC. The study did not meet the requirements for formal ethics board review as defined using the National Health Service (NHS) Health Research Authority research decision tool (http://www.hra-decisiontools.org.uk/research/).

The study was conducted following the RECORD (REporting of studies Conducted using Observational Routinely collected Data) guidelines.^30^

## 3 RESULTS

The study population consisted of 3,851,055 children and adults with valid clinical data and no history of eczema prior to 01/01/2009 (Flowchart S1). A total of 174,606 people developed incident eczema over the study period.

### 3.1 Peak incidence of eczema: younger than one and older than 80 years

**FIGURE 1:**
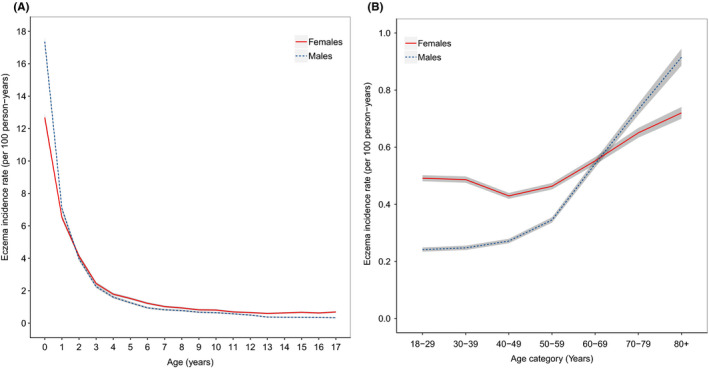
The crude incidence of eczema per 100 person‐years by age and sex in (A) children (*n* = 913,606) and (B) adults (*n* = 3,149,160). Grey shading represents 95% confidence intervals

In children, the incidence of eczema is highest in male infants, with a peak incidence of 17.4 (95% CI 17.1, 17.6) per 100 person‐years in infants younger than one year (Figure 1A, Table S1). From age 2 onwards, the incidence is higher in females than males and falls progressively up to the age of seven for both sexes, after which incidence plateaus up to age 18. In adults, incidence is relatively stable from ages 18–49, after which there is a steady increase in incidence for both sexes (Figure 1B, Table S1). This increase is most marked in males, resulting in a greater incidence of eczema in males compared to females from age 70. Over 2009–2018, we observed a gradual decrease in the incidence of eczema in both adults and children (Figure S1).

### 3.2 Sociodemographic factors associated with incident eczema vary by age group

In infants, the incidence of eczema recorded in primary care was higher in those of higher socio‐economic status (IMD quintiles 4 and 5), but in those older than two years of age the trend was reversed and persisted throughout adulthood (Table 1). Compared with people of white ethnicity, across the lifespan people of Asian ethnicity have a higher incidence of eczema. People of black and, to a lesser extent, mixed ethnicity also have a higher incidence of eczema than people of white ethnicity, up to age 50. A higher incidence was observed in urban than rural areas for all age groups (Table 1).

**TABLE 1:** Adjusted incidence rate ratios of new‐onset eczema by age category and sociodemographic characteristics, 2009–2018 inclusive in children (*n* = 913,606) and adults (*n* = 3,149,160)^a^

	Age < 2	Age 2–11	Age 12–17	Age 18–49	Age 50+
IMD quintile^b^					
1 (most deprived)	1.00 (ref)	1.00 (ref)	1.00 (ref)	1.00 (ref)	1.00 (ref)
2	1.00 (0.96, 1.03)	0.99 (0.96, 1.02)	0.97 (0.89, 1.04)	0.88 (0.85, 0.91)**	0.98 (0.95, 1.02)
3	0.97 (0.93, 1.00)^*^	0.96 (0.93, 0.99)^*^	0.90 (0.83, 0.97)^*^	0.87 (0.84, 0.90)**	0.94 (0.91, 0.97)**
4	1.05 (1.01, 1.08)^*^	0.99 (0.96, 1.02)	0.89 (0.82, 0.96)^*^	0.87 (0.84, 0.90)**	0.93 (0.90, 0.96)**
5 (least deprived)	1.06 (1.03, 1.09)**	0.96 (0.93, 0.99)^*^	0.89 (0.83, 0.96)^*^	0.89 (0.86, 0.92)**	0.95 (0.92, 0.98)^*^
Ethnicity^c^					
White	1.00 (ref)	1.00 (ref)	1.00 (ref)	1.00 (ref)	1.00 (ref)
Asian	1.68 (1.62, 1.74)**	1.32 (1.28, 1.37)**	1.65 (1.52, 1.80)**	1.53 (1.48, 1.58)**	1.62 (1.55, 1.69)**
Black	1.69 (1.61, 1.78)**	1.31 (1.25, 1.38)**	1.31 (1.16, 1.48)**	1.18 (1.12, 1.24)**	0.81 (0.75, 0.87)**
Mixed	1.28 (1.21, 1.36)**	1.08 (1.02, 1.15)^*^	1.19 (0.99, 1.41)	1.13 (1.03, 1.23)^*^	0.96 (0.83, 1.10)
Other	1.08 (0.97, 1.19)	0.87 (0.79, 0.96)^*^	0.95 (0.74, 1.19)	1.00 (0.91, 1.10)	0.98 (0.85, 1.13)
Rural–urban classification^d^					
Rural	1.00 (ref)	1.00 (ref)	1.00 (ref)	1.00 (ref)	1.00 (ref)
Urban	1.10 (1.07, 1.13)**	1.11 (1.08, 1.14)**	1.09 (1.02, 1.16)^*^	1.05 (1.02, 1.09)**	1.10 (1.07, 1.12)**

Abbreviation: IMD, index of multiple deprivation.

^a^ Models additionally adjusted for sex within each age category.

^b^ IMD data were not available for *n* = 81,539.

^c^ Ethnicity data were not available for *n* = 985,732.

^d^ Rural–urban classification was not available for *n* = 78,214.

** *p* < .001. *p < .05.

### 3.3 Active eczema has a bimodal age distribution

**FIGURE 2:**
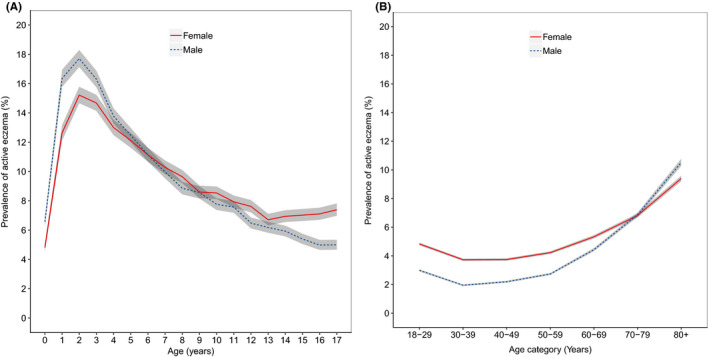
Prevalence of eczema by age category and sex in (A) children (*n* = 570,536) and (B) adults (*n* = 2,168,805). Grey shading represents 95% confidence intervals

The prevalence of active eczema is greatest in children aged 1–4 and then decreases with increasing age with a nidus in the fourth and fifth decades of life (Figure 2, Table S2). Active eczema then increases again in prevalence with increasing age, almost returning to the peak childhood prevalence in those aged 80 years and older. Over the decade we studied, we found a slight decrease in prevalence of active eczema in children but little change in prevalence in adults (Figure S2).

### 3.4 Factors associated with active eczema vary by age

In children, overall prevalence of active eczema is similar in males and females, but in adults, active eczema is more prevalent in females (Tables 2 and 3). In children and adults, active eczema is more prevalent in those of Asian, black, and mixed ethnicity than in those of white ethnic background. Across the lifespan, a higher prevalence is also found in the most deprived IMD quintile compared with all other IMD quintiles. In addition, a higher prevalence is found in urban than rural areas across the lifespan (Tables 2 and 3).

**TABLE 2:** Prevalence of active eczema by sociodemographic factors in children

	eczema cases (*n*)	Denominator	Prevalence (%)	Unadjusted odds ratio	Adjusted odds ratio
Overall	54,659	570,536	9.6	NA	NA
Sex					
Female	26,571	278,131	9.6	1.00 (ref)	1.00 (ref)
Male	28,088	292,405	9.6	1.01 (0.99, 1.02)	1.01 (0.99, 1.03)
Age category^a^					
<2	4532	56,725	8.0	1.00 (ref)	1.00 (ref)
2–6	23,025	162,224	14.2	1.90 (1.87, 1.94)**	1.89 (1.86, 1.93)**
7–11	15,232	167,450	9.1	1.15 (1.12, 1.19)**	1.13 (1.09, 1.16)**
12–17	11,870	184,127	6.4	0.79 (0.76, 0.83)**	0.78 (0.74, 0.82)**
IMD quintile^b^					
1 (most deprived)	11,409	101,632	11.2	1.00 (ref)	1.00 (ref)
2	10,396	98,479	10.6	0.93 (0.91, 0.96)**	0.96 (0.93, 0.98)^*^
3	9330	104,248	8.9	0.78 (0.75, 0.81)**	0.90 (0.87, 0.93)**
4	10,273	116,432	8.8	0.77 (0.74, 0.79)**	0.92 (0.89, 0.95)**
5 (least deprived)	12,538	140,487	8.9	0.77 (0.75, 0.80)**	0.95 (0.92, 0.97)**
Ethnicity^c^					
White	25,061	297,146	8.4	1.00 (ref)	1.00 (ref)
Asian	6602	40,165	16.4	2.14 (2.11, 2.16)**	2.10 (2.07, 2.13)**
Black	3247	19,694	16.5	2.14 (2.10, 2.18)**	2.13 (2.09, 2.17)**
Mixed	1825	14,452	12.6	1.57 (1.52, 1.62)**	1.49 (1.44, 1.55)**
Other	558	6647	8.4	0.99 (0.91, 1.08)	0.98 (0.89, 1.07)
Rural–urban^d^ Classification^a^					
Rural	8968	113,361	7.9	1.00 (ref)	1.00 (ref)
Urban	45,030	448,528	10.0	1.30 (1.28, 1.32)**	1.11 (1.08, 1.13)**

Note

Derived using data from 2018.

Abbreviation: IMD, index of multiple deprivation.

**FIGURE 3:**
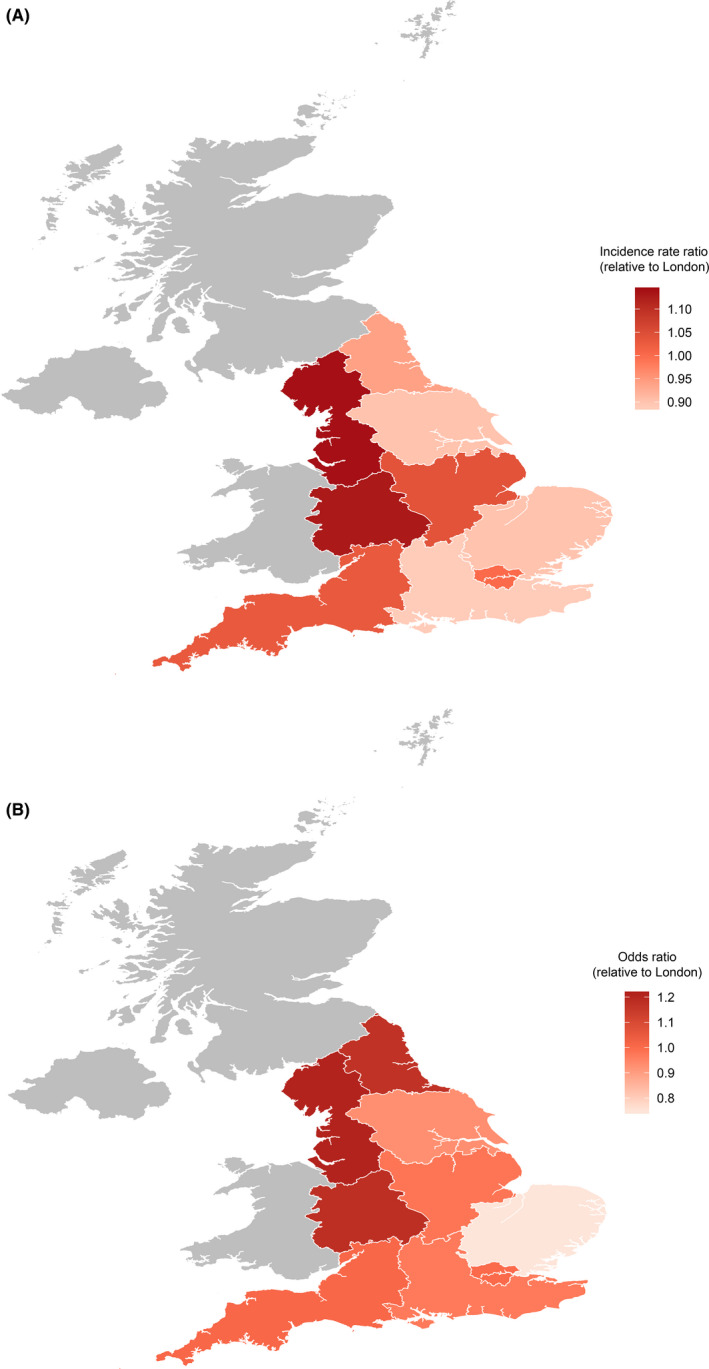
The geographical distribution of eczema in England. (A) Adjusted incidence rate ratios (aIRR) for eczema in 2018 by geographical region (*n* = 2,336,322). aIRR are relative to London. (B) Adjusted odds ratios (OR) for active eczema in 2018 by geographical region (*n* = 2,742,094). OR are relative to London

^a^ Additional age category split (2–6, 7–11) was added post hoc due to the marked change in disease prevalence across this age group (Figure 3A).

^b^ IMD data were not available for *n* = 9258.

^c^ Ethnicity data were not available for *n* = 192,432.

^d^ Rural–urban classification was not available for *n* = 8647.

** *p* < .001. *p < .01.

**TABLE 3:** Prevalence of active eczema by sociodemographic factors in adults

	Cases (*n*)	Denominator	Prevalence (%)	Unadjusted odds ratio	Adjusted odds ratio
Overall	93,323	2,168,805	4.3	NA	NA
Sex					
Female	54,681	1,093,890	5.0	1.00 (ref)	1.00 (ref)
Male	38,642	1,074,915	3.6	0.71 (0.70, 0.72)**	0.74 (0.73, 0.76)**
Age category					
18–29	16,234	415,834	3.9	1.00 (ref)	1.00 (ref)
30–39	10,833	383,437	2.8	0.72 (0.69, 0.74)**	0.70 (0.68, 0.73)**
40–49	10,571	358,318	3.0	0.75 (0.72, 0.77)**	0.75 (0.73, 0.78)**
50–59	12,676	365,631	3.5	0.88 (0.86, 0.91)**	0.92 (0.89, 0.94)**
60–69	13,659	279,676	4.9	1.26 (1.24, 1.29)**	1.31 (1.29, 1.34)**
70–79	15,214	222,425	6.8	1.81 (1.78, 1.83)**	1.89 (1.86, 1.91)**
80+	14,136	143,484	9.9	2.69 (2.67, 2.71)**	2.77 (2.75, 2.80)**
IMD quintile^a^					
1 (most deprived)	15,012	328,960	4.6	1.00 (ref)	1.00 (ref)
2	15,086	362,050	4.2	0.91 (0.89, 0.93)**	0.90 (0.88, 0.92)**
3	17,086	412,159	4.1	0.90 (0.88, 0.93)**	0.88 (0.85, 0.90)**
4	21,111	481,242	4.4	0.96 (0,94, 0.98)**	0.91 (0.89, 0.93)**
5 (least deprived)	23,889	540,802	4.4	0.97 (0.95, 0.99)^*^	0.90 (0.88, 0.93)**
Ethnicity^b^					
White	62,587	1,426,211	4.4	1.00 (ref)	1.00 (ref)
Asian	8354	148,387	5.6	1.30 (1.28, 1.32)**	1.58 (1.55, 1.60)**
Black	2546	59,354	4.3	0.98 (0.94, 1.02)	1.14 (1.10, 1.18)**
Mixed	981	24,484	4.0	0.91 (0.84, 0.97)^*^	1.12 (1.05, 1.18)**
Other	676	22,753	3.0	0.67 (0.59, 0.74)**	0.83 (0.76, 0.91)**
Rural–Urban^c^ Classification^a^					
Rural	19,996	462,375	4.3	1.00 (ref)	1.00 (ref)
Urban	72,295	1,664,907	4.3	1.00 (0.99, 1.02)	1.04 (1.03, 1.06)**

Note

Derived using data from 2018.

Abbreviation: IMD, index of multiple deprivation.

^a^ IMD data were not available for *n* = 43,592.

^b^ Ethnicity data were not available for *n* = 487,616.

^c^ Rural–Urban classification was not available for *n* = 41,523.

** *p* < .001. *p < .01.

### 3.5 Eczema incidence and prevalence are highest in the North‐West and West of England

The crude incidence of eczema is highest in the North‐West, London, and the West Midlands across the lifespan (Table S3). After adjustment for age, sex, SES, and ethnicity, the higher incidence in the North‐West becomes even more pronounced (Figure 3A and Table S4). Similarly, the highest prevalence rates for active eczema are in London, the North‐West, and the West Midlands (Table S5), even after adjustment for confounding factors (Figure 3B and Table S6). When analysed separately in children and adults, incidence and prevalence are highest in the North‐West and West for both groups (Tables S4 and S6).

## 4 DISCUSSION

In a large population‐based cohort of more than 3.85 million people in England, we found that the incidence and prevalence of eczema had a bimodal age distribution. The highest incidence of eczema was seen in children younger than one (incidence rate 15.0 per 100 person‐years), with a steady decrease during childhood and early adulthood and a subsequent steady increase after age 50. We also observed striking differences in the incidence and prevalence of eczema by sex, socio‐economic status, ethnicity, and geography.

### 4.1 Comparison with other studies

In agreement with our study, a recent UK analysis of children in primary care found the highest rates of incident eczema in infancy but that eczema was also common across childhood.^16^ To the best of our knowledge, our study is the first to provide population‐based UK data on eczema incidence in adults. Our data for prevalent eczema in children are comparable with a smaller cross‐sectional analysis of patients from four general practices in 1998 that found the highest prevalence of disease (22%) in children aged 1–2 years, and a recent longitudinal study, conducted by Abuabara et al, that used data from the 1958 and 1970 British cohort studies to estimate an overall AE prevalence of 7%–14% in childhood.^31^ Our reported prevalence in adults (4.3%) is likely lower than that reported by Abuabara et al (5%–12%) due to the different time period and sampling strategies used as well as our case definition, as we limited prevalent cases to those with active disease.^15^ The decrease in eczema incidence and prevalence throughout childhood and adulthood may be partly due to maturation of the skin barrier properties.^32^, ^33^, ^34^ A gradual decline in water holding properties of the skin barrier in older age would also be an explanation for the second peak in eczema incidence seen in older adults, but this needs further investigation.^35^, ^36^, ^37^

### 4.2 Sex differences

The notable differences in the incidence of childhood eczema by sex, with an increased incidence in male infants younger than 2 and female children thereafter, are in concordance with a recent study using a different UK primary care dataset and previous Scandinavian population‐based studies.^16^, ^38^, ^39^ Comparable differences by sex have also been seen in the childhood prevalence of allergic rhinitis and asthma during childhood.^40^ In adults, the increased incidence observed in older males compared with females was previously reported in Japanese hospital‐based patients,^32^, ^41^ but to our knowledge not in a population‐based setting.

### 4.3 Socio‐economic status differences

Previous studies evaluating the relationship between atopic conditions and SES have, in general, suggested a higher prevalence of eczema in less deprived SES groups.^42^ In contrast, we found a higher incidence of eczema in less deprived SES groups (IMD quintiles 4 and 5) only in infants, a finding consistent with other recent UK primary care‐based data.^16^ Across the rest of the lifespan, less deprived SES was consistently associated with a lower rate of incident eczema. Differences with previous studies may relate to variation in setting and in methodology, with a particular strength of this analysis being the comprehensive adjustment for other sociodemographic factors and geography.

### 4.4 Ethnicity differences

Consistent with our study, US data suggest that people of Asian and black ethnicity are substantially more likely to attend medical services for eczema than people of white ethnicity.^43^ A greater prevalence of eczema in black and Asian children has also been reported in another large US database study.^44^ Genetic, skin barrier, immune, and environmental differences may underlie the increased eczema risk in people of different ethnicities. For example, population genetics studies have identified three filaggrin mutations in East Asian eczema populations that are not present in the white European eczema population.^45^ Unique filaggrin loss‐of‐function mutations have also been identified in black children with eczema.^46^

### 4.5 Geographical distribution

The only previous data on the geographical distribution of eczema in England are the 1958 British Cohort Study, which identified the North Midlands, Eastern region, London, and Southern region, as areas of higher prevalence.^47^ Direct comparisons with our results are limited by differences in geographical boundaries, socio‐economic changes, and differences in the population samples studied. Variation in eczema prevalence by geographical area has also been seen in other countries.^44^ The environmental factors that increase the risk for eczema have yet to be fully elucidated, but ultraviolet radiation exposure, lower air temperature, and higher use of indoor central heating have all been linked to higher eczema rates,^48^, ^49^ and this offers a potential explanation for the higher rates seen in North‐West England. Consistent with our study, a higher incidence and prevalence of eczema in urban areas have been seen in previous studies of eczema world‐wide^44^, ^48^, ^50^ and has been linked to differences in air pollution and heavy road traffic.^2^, ^51^, ^52^ A similar pattern has also been seen in the distribution of allergic rhinitis.^53^

### 4.6 Strengths and limitations

Key strengths of this study include our use of a large primary care network to capture eczema diagnoses using a previously validated algorithm. The distribution of the network and variety of the contributing GP practices has enabled us to provide novel insight into geographical and urban–rural variation in eczema. Our high level of data capture on SES and ethnicity in this large population and the fact that the majority of eczema treatment is undertaken in primary care in the UK are also important strengths.

Several limitations are worth noting. First, a diagnosis of eczema requires presentation to primary care, and we will therefore have missed minor and subclinical disease, leading to an underestimate of eczema incidence and prevalence. We were unable to determine whether this issue has a differential effect by sex and ethnicity. Similarly, we cannot be certain that the first recorded eczema diagnosis in the primary care record is an accurate reflection of initial disease onset in all cases, although this was mitigated by our study design which excluded individuals with an eczema diagnosis within 1 year of registering with a general practice. Second, our case definition requires prescriptions for eczema treatments, some of which are available over the counter. People purchasing all their treatments directly from a pharmacy will therefore be missed by our approach. We were unable to examine the association between familial history of atopic or allergic disease and eczema onset, as this is not well captured in UK routine primary care records. Finally, more comprehensive validation of the eczema diagnostic algorithm and validation of the active eczema case definition would be of interest for further studies, particularly in older age groups where clinical coding may not allow eczema to be distinguished from other types of dermatitis.

### 4.7 Implications of the findings

Our results provide timely information on the epidemiology of eczema in the UK and highlight the need for additional studies to more fully understand the pathogenesis of eczema, as well we environmental and ethnicity‐related factors that may drive differences in disease burden. In particular, more in‐depth evaluation of variation in incidence rates of eczema across different ethnic groups, for example with stratification by age and sex within ethnic groups, would be of considerable importance for future work. Corroborations of our findings in other populations would also be of great interest, as would investigation of the association between AD incidence and prevalence and environmental factors such as climate. Furthermore, our study suggests many well‐used diagnostic criteria for eczema may require refinement given their inclusion of early age of onset in the diagnosis,^54^ as use of this definition will exclude the large number of cases of adult‐onset eczema we identified. It will also be important to examine the causes of true adult‐onset eczema, as this may be genetically and immunologically distinct from eczema that starts in earlier life.

## 5 CONCLUSIONS

In summary, our study uses a large English primary care database to show that eczema is not just a condition of childhood, highlighting a bimodal age distribution of disease with peaks in infants and older adults. There are considerable differences in eczema incidence and prevalence by ethnicity, sociodemographic characteristics, and geography, demonstrating the need to consider these factors when assessing health needs.

## CONFLICTS OF INTEREST

S. de Lusignan is Director of the Royal College of General Practitioners Research and Surveillance Centre as part of his academic post; he has also received funding for projects from Eli Lilly, AstraZeneca, GSK, Seqirus, and Takeda, all through his universities and none related to this study. C. Feeney is an employee of Pfizer. J. Dennis and A. McGovern are employees of Momentum Data who were paid consultants to Pfizer in connection with the development of this manuscript. C. Flohr is chief investigator of the UK National Institute for Health Research—funded TREAT (ISRCTN15837754) and SOFTER (ClinicalTrials.gov: NCT03270566) trials and the UK‐Irish Atopic Eczema Systemic Therapy Register (A‐STAR; ISRCTN11210918) and is a principal investigator in the European Union Horizon 2020—funded BIOMAP Consortium (http://www.biomap-imi.eu/). His department has also received funding from Sanofi‐Genzyme. All other authors have no competing interests to declare.

## AUTHOR CONTRIBUTION

C. Flohr, C. Feeney, H. Alexander, A. McGovern, J. Dennis, and S. de Lusignan developed the study concept and design. S. de Lusignan, C. Feeney, H. Alexander, C. Broderick, and C. Flohr performed the study and wrote the paper. J. Dennis and A. McGovern conducted and are responsible for the data analysis. All authors critically reviewed the manuscript. S. de Lusignan had full access to all the data in the study and takes responsibility for the integrity of the data and the accuracy of the data analysis.

## Supporting information

Appendix S1

## Data Availability

The RCGP RSC dataset is held securely at University of Oxford and the University of Surrey and can be accessed by bone fide researchers. Approval is on a project‐by‐project basis (www.rcgp.org.uk/rsc). Ethical approval by an NHS Research Ethics Committee may be needed before any data release/other appropriate approval. Researchers wishing to directly analyse the patient‐level pseudonymized data will be required to complete information governance training and work on the data from university secure servers. Patient‐level data cannot be taken out of the secure network.
