# Supplementary material for: The epidemiology of eczema in children and adults in England: A population‐based study using primary care data
Source: Clin Exp Allergy. 2020 Nov 26;51(3):471–82. doi: 10.1111/cea.13784 (PMC7984097; doi:10.1111/cea.13784)
Supplement: Supplementary file 1 — Appendix S1 [file CEA-51-471-s001.pdf]

Supplementary material for:

The epidemiology of atopic dermatitis in children and adults: population-based cohort study

Authors: Simon de Lusignan, Helen Alexander, Conor Broderick, John Dennis, Andrew McGovern, Claire Feeney, Carsten Flohr

eFlowchart 1: Flowchart for construction of study population

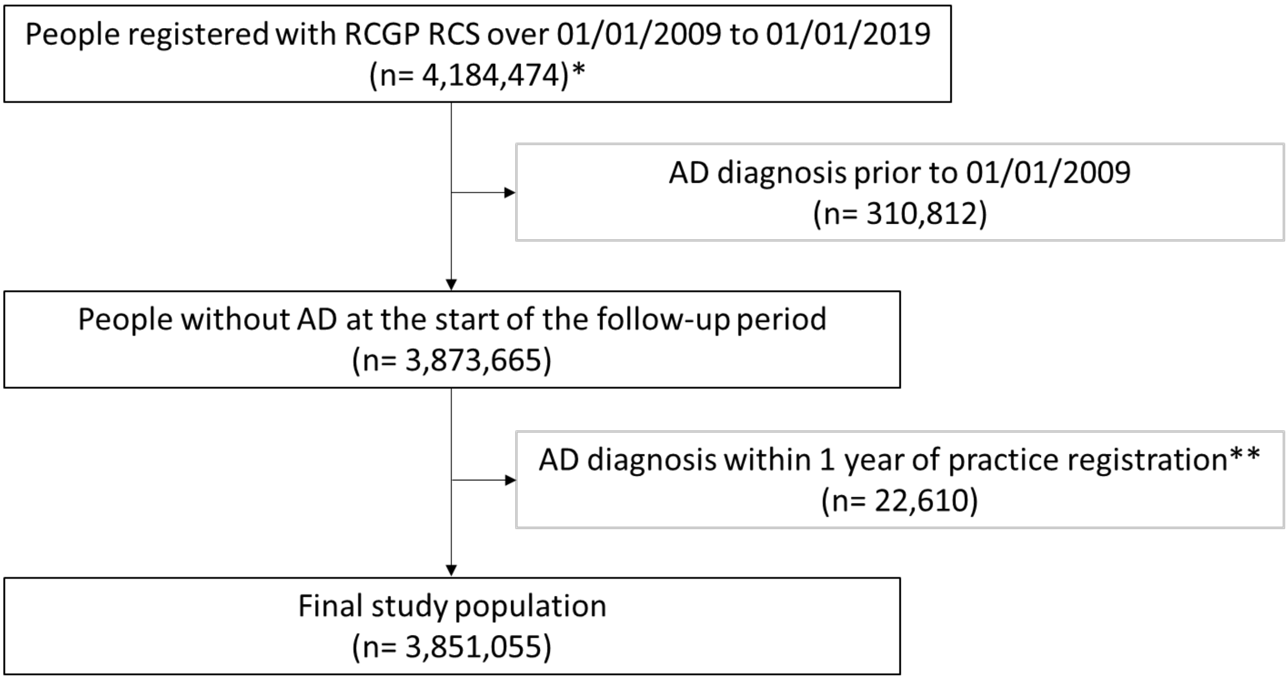

* Final study population for analysis of AD prevalence
** Not applicable if less than one year old

eFigure 1: Incidence rates of AD by calendar year, 2009-2018 in (A) children (n=913,606) and (B) adults (n=3,149,160). Grey shading represents 95% confidence intervals.

(A) Children

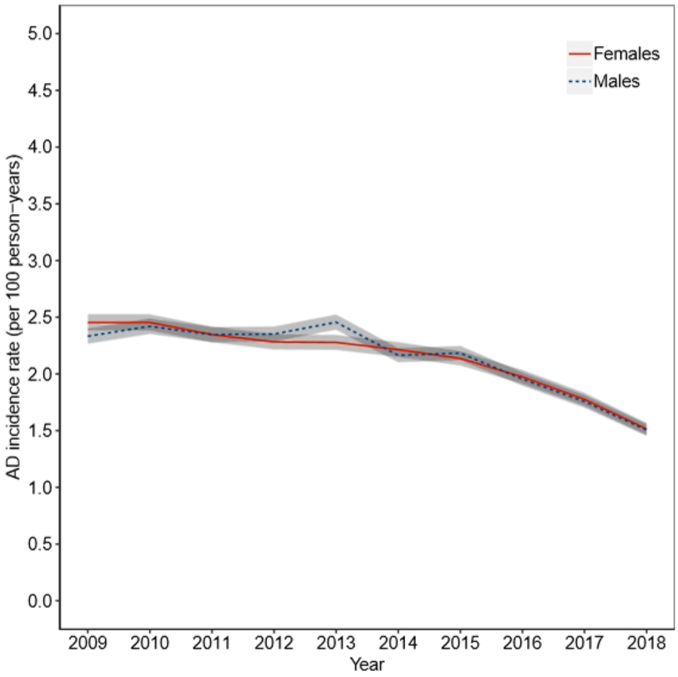

(B) Adults

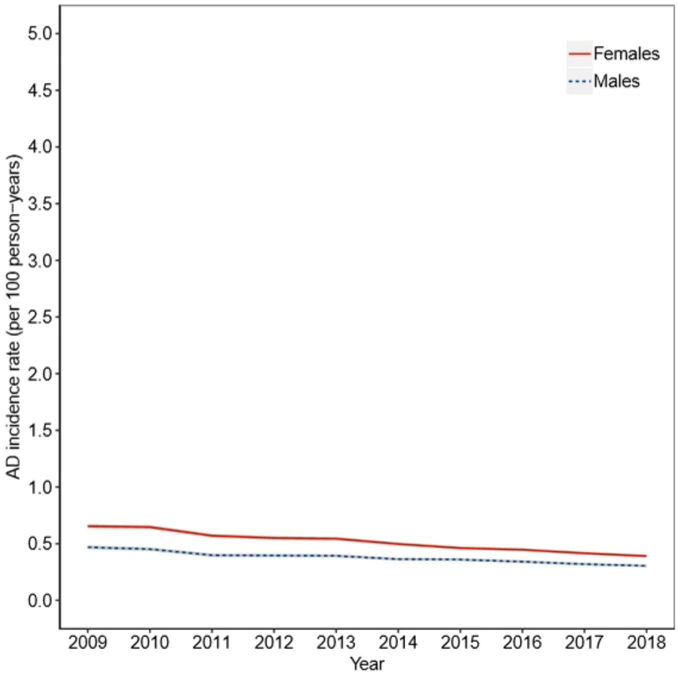

eFigure 2: The prevalence of active AD, by calendar year in (A) children and (B) adults. Grey shading represents 95% confidence intervals.

(A) Children

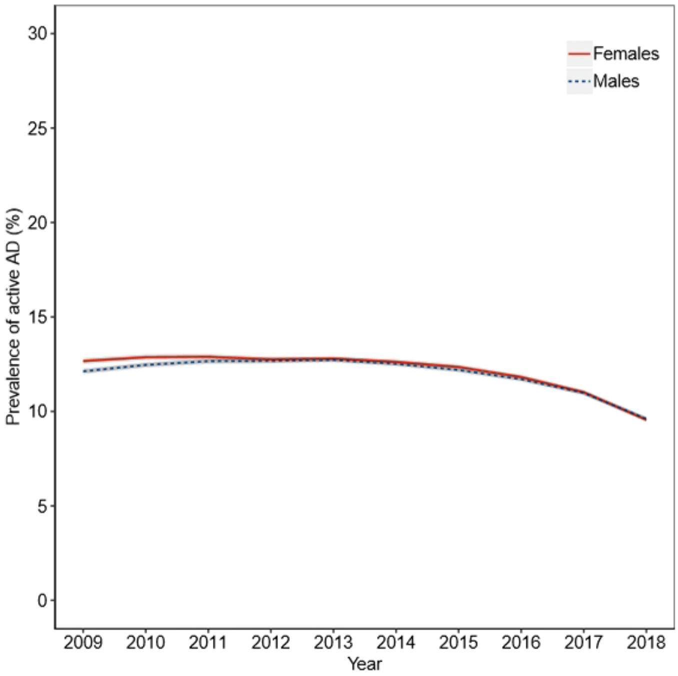

B) Adults

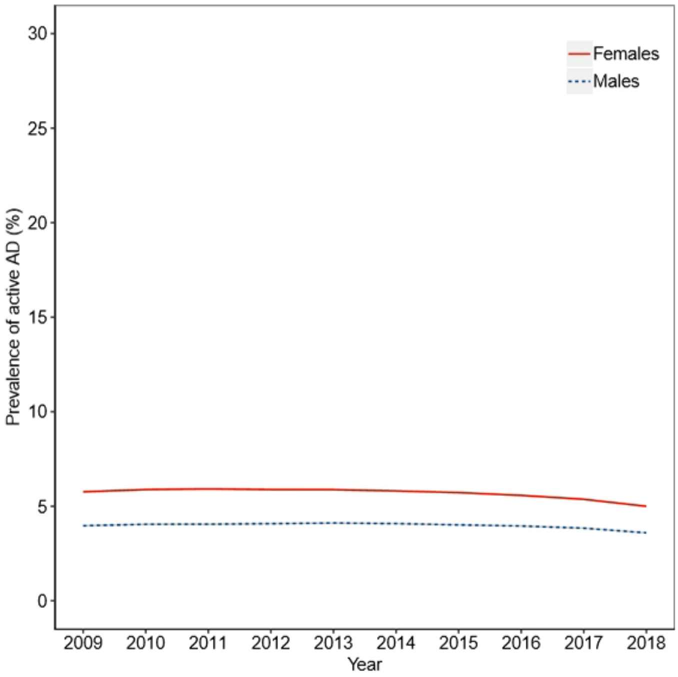

eTable 1: The incidence of AD per 100-person years, by age and sex in (A) children (n=913,606) and (B) adults (n=3,149,160)

(A) Children

	Incidence rate (95% CI)		
	**Overall**	**Males**	**Females**
**Age**			
**0**	15.04 (14.87, 15.22)	17.36 (17.1, 17.62)	12.68 (12.46, 12.91)
**1**	6.79 (6.69, 6.90)	7.06 (6.92, 7.21)	6.52 (6.38, 6.67)
**2**	4.05 (3.97, 4.13)	3.96 (3.85, 4.08)	4.13 (4.02, 4.25)
**3**	2.35 (2.29, 2.41)	2.25 (2.17, 2.34)	2.44 (2.35, 2.53)
**4**	1.69 (1.64, 1.75)	1.60 (1.53, 1.67)	1.79 (1.71, 1.86)
**5**	1.39 (1.34, 1.44)	1.26 (1.20, 1.33)	1.52 (1.45, 1.59)
**6**	1.08 (1.04, 1.12)	0.94 (0.89, 1.00)	1.22 (1.16, 1.29)
**7**	0.92 (0.88, 0.96)	0.82 (0.77, 0.88)	1.02 (0.96, 1.08)
**8**	0.85 (0.82, 0.89)	0.78 (0.72, 0.83)	0.94 (0.88, 1.00)
**9**	0.74 (0.71, 0.78)	0.67 (0.63, 0.72)	0.82 (0.77, 0.88)
**10**	0.72 (0.69, 0.76)	0.65 (0.60, 0.69)	0.81 (0.75, 0.86)
**11**	0.63 (0.60, 0.67)	0.57 (0.53, 0.62)	0.69 (0.64, 0.74)
**12**	0.57 (0.54, 0.61)	0.50 (0.46, 0.54)	0.65 (0.61, 0.70)
**13**	0.48 (0.45, 0.51)	0.38 (0.34, 0.41)	0.60 (0.55, 0.64)
**14**	0.49 (0.46, 0.52)	0.36 (0.33, 0.40)	0.63 (0.59, 0.68)
**15**	0.50 (0.48, 0.53)	0.36 (0.33, 0.39)	0.67 (0.62, 0.72)
**16**	0.48 (0.45, 0.51)	0.35 (0.32, 0.38)	0.63 (0.58, 0.68)
**17**	0.50 (0.47, 0.53)	0.33 (0.30, 0.36)	0.69 (0.64, 0.74)

B) Adults

	Incidence rate (95% CI)		
	**Overall**	**Males**	**Females**
**Age**			
**18-29**	0.36 (0.36, 0.37)	0.24 (0.23, 0.25)	0.49 (0.48, 0.50)
**30-39**	0.36 (0.36, 0.37)	0.25 (0.24, 0.26)	0.49 (0.48, 0.50)
**40-49**	0.35 (0.34, 0.35)	0.27 (0.26, 0.28)	0.43 (0.42, 0.44)
**50-59**	0.40 (0.39, 0.41)	0.35 (0.34, 0.35)	0.46 (0.45, 0.47)
**60-69**	0.55 (0.54, 0.56)	0.54 (0.53, 0.56)	0.55 (0.54, 0.57)
**70-79**	0.69 (0.68, 0.70)	0.73 (0.71, 0.75)	0.65 (0.63, 0.67)
**80+**	0.79 (0.78, 0.81)	0.92 (0.89, 0.94)	0.72 (0.70, 0.74)

eTable 2: Prevalence of atopic dermatitis (AD), by age category and sex in (A) children (n=570,536) and (B) adults (n=2,168,805). Derived using data from 2018.

(A) Children

	Prevalence (%) with 95% CIs		
	**Overall**	**Males**	**Females**
**Age**			
**0**	5.7 (5.5, 5.9)	6.6 (6.2, 6.9)	4.8 (4.5, 5.1)
**1**	14.5 (14.1, 14.9)	16.4 (15.8, 16.9)	12.6 (12.1, 13.2)
**2**	16.5 (16.1, 16.9)	17.7 (17.1, 18.3)	15.2 (14.7, 15.8)
**3**	15.5 (15.1, 15.9)	16.3 (15.7, 16.9)	14.7 (14.1, 15.2)
**4**	13.4 (13.0, 13.8)	13.8 (13.3, 14.3)	13.0 (12.5, 13.5)
**5**	12.3 (12.0, 12.7)	12.5 (12.0, 13.0)	12.1 (11.6, 12.6)
**6**	11.1 (10.8, 11.5)	11.1 (10.7, 11.6)	11.1 (10.7, 11.6)
**7**	10.1 (9.8, 10.4)	10 (9.5, 10.4)	10.3 (9.8, 10.8)
**8**	9.2 (8.9, 9.6)	8.9 (8.4, 9.3)	9.6 (9.2, 10.1)
**9**	8.6 (8.3, 8.9)	8.6 (8.2, 9.0)	8.6 (8.2, 9.0)
**10**	8.1 (7.8, 8.4)	7.8 (7.4, 8.2)	8.5 (8.1, 9.0)
**11**	7.7 (7.5, 8.0)	7.6 (7.2, 8.0)	7.9 (7.5, 8.4)
**12**	7.0 (6.7, 7.3)	6.5 (6.1, 6.9)	7.6 (7.2, 8.0)
**13**	6.4 (6.2, 6.7)	6.2 (5.8, 6.6)	6.7 (6.3, 7.1)
**14**	6.4 (6.2, 6.7)	5.9 (5.6, 6.3)	6.9 (6.5, 7.4)
**15**	6.2 (5.9, 6.5)	5.0.4 (5, 5.8)	7.0 (6.6, 7.4)
**16**	6.0 (5.7, 6.3)	5.0 (4.6, 5.3)	7.1 (6.7, 7.5)
**17**	6.2 (5.9, 6.4)	5.0 (4.7, 5.3)	7.4 (7.0, 7.8)

B) Adults

	Prevalence (%) with 95% CIs		
	**Overall**	**Males**	**Females**
**Age**			
**18-29**	3.9 (3.8, 4.0)	3.0 (2.9, 3.1)	4.8 (4.7, 4.9)
**30-39**	2.8 (2.8, 2.9)	1.9 (1.9, 2.0)	3.7 (3.6, 3.8)
**40-49**	3.0 (2.9, 3.0)	2.2 (2.1, 2.3)	3.7 (3.7, 3.8)
**50-59**	3.5 (3.4, 3.5)	2.7 (2.7, 2.8)	4.2 (4.1, 4.3)
**60-69**	4.9 (4.8, 5.0)	4.4 (4.3, 4.5)	5.3 (5.2, 5.4)
**70-79**	6.8 (6.7, 6.9)	6.9 (6.7, 7.0)	6.8 (6.7, 7.0)
**80+**	9.9 (9.7, 10.0)	10.5 (10.3, 10.8)	9.4 (9.2, 9.6)

eTable 3: Incidence of AD by geographical region of England, 2018 (per 100 person-years) with 95% confidence intervals (n=2,336,322)

	Age category				
	<2	2-11	12-17	18-49	50+
East Midlands	6.8 (5.6-8.1)	1.4 (1.2-1.6)	0.4 (0.3-0.6)	0.2 (0.2-0.3)	0.5 (0.4-0.6)
East of England	5.4 (4.3-6.8)	1.0 (0.8-1.2)	0.5 (0.4-0.7)	0.2 (0.2-0.3)	0.4 (0.4-0.5)
London	9.2 (8.5-9.9)	1.5 (1.4-1.6)	0.5 (0.4-0.6)	0.3 (0.2-0.3)	0.5 (0.4-0.5)
North East	5.3 (3.9-6.9)	1.3 (1.0-1.6)	0.3 (0.2-0.6)	0.3 (0.2-0.3)	0.4 (0.4-0.5)
North West	9.7 (8.8-10.5)	1.5 (1.4-1.6)	0.4 (0.3-0.5)	0.3 (0.2-0.3)	0.5 (0.5-0.6)
South East	6.5 (5.8-7.1)	1.1 (1.0-1.2)	0.3 (0.2-0.3)	0.3 (0.2-0.3)	0.4 (0.4-0.4)
South West	6.3 (5.5-7.1)	1.3 (1.2-1.5)	0.4 (0.3-0.5)	0.3 (0.2-0.3)	0.5 (0.4-0.5)
West Midlands	8.5 (7.4-9.8)	1.5 (1.3-1.6)	0.3 (0.2-0.5)	0.3 (0.2-0.3)	0.5 (0.5-0.6)
Yorkshire and The Humber	6.5 (5.7-7.4)	1.3 (1.1-1.4)	0.4 (0.3-0.5)	0.2 (0.2-0.3)	0.4 (0.3-0.4)

eTable 4: Adjusted incidence rate ratios (IRR) with 95% CIs for AD by geographical region, 2018 (n=2,336,322). Model adjusted for age category, sex, ethnicity, index of multiple deprivation, and rural/urban classification. ORs are relative to London.

	Overall IRR (95% CI)	Children <18 IRR (95% CI)	Adults ≥ 18 IRR (95% CI)
East Midlands	1.04 (0.95-1.13)	1.01 (0.88. 1.14)	1.10 (0.96, 1.23)
East of England	0.90 (0.80-0.99)	0.82 (0.68. 0.97)	1.00 (0.86. 1.13)
London	1.00 (ref)	1.00 (ref)	1.00 (ref)
North East	0.94 (0.83-1.06)	0.84 (0.67, 1.01)	1.07 (0.92. 1.22)
North West	1.14 (1.08-1.20)	1.11 (1.03. 1.19)	1.20 (1.11, 1.28)
South East	0.89 (0.83-0.96)	0.83 (0.74. 0.92)	1.00 (0.91, 1.09)
South West	1.03 (0.96-1.10)	0.96 (0.86. 1.05)	1.15 (1.05. 1.25)
West Midlands	1.13 (1.06-1.21)	1.05 (0.95. 1.16)	1.25 (1.14, 1.36)
Yorkshire and The Humber	0.90 (0.82-0.97)	0.90 (0.80. 1.00)	0.93 (0.82. 1.04)

eTable 5: Prevalence of active AD by geographical region of England, 2018 (n=2,742,094)

	Age category				
	<2	2-11	12-17	18-49	50+
**East Midlands**	7%	10%	6%	3%	5%
**East of England**	6%	9%	5%	2%	4%
**London**	9%	15%	9%	3%	6%
**North East**	6%	12%	7%	4%	6%
**North West**	9%	13%	7%	4%	6%
**South East**	7%	10%	6%	3%	5%
**South West**	6%	10%	6%	3%	5%
**West Midlands**	8%	12%	7%	4%	6%
**Yorkshire and The Humber**	6%	10%	6%	3%	5%

eTable 6: Adjusted odds ratios (OR) with 95% CIs for active AD in 2018 by geographical region (n=2,742,094). Model adjusted for age category, sex, ethnicity, index of multiple deprivation, and rural/urban classification. ORs are relative to London.

	Overall<br>OR (95% CI)	Children <18<br>OR (95% CI)	Adults ≥ 18<br>OR (95% CI)
**East Midlands**	1.00 (0.97, 1.02)	0.88 (0.83, 0.92)	1.10 (1.06, 1.14)
**East of England**	0.77 (0.73. 0.80)	0.70 (0.64, 0.75)	0.83 (0.79, 0.87)
**London**	1.00 (ref)	1.00 (ref)	1.00 (ref)
**North East**	1.18 (1.15, 1.21)	1.03 (0.97, 1.08)	1.31 (1.27, 1.35)
**North West**	1.21 (1.19, 1.23)	1.04 (1.01, 1.07)	1.35 (1.33, 1.37)
**South East**	0.97 (0.95, 0.99)	0.83 (0.80, 0.87)	1.09 (1.07, 1.12)
**South West**	1.02 (1.00, 1.04)	0.85 (0.81, 0.89)	1.17 (1.14, 1.20)
**West Midlands**	1.20 (1.17, 1.22)	1.02 (0.98, 1.06)	1.34 (1.31, 1.37)
**Yorkshire and The Humber**	0.93 (0.91, 0.96)	0.86 (0.82, 0.89)	1.01 (0.98, 1.04)
